# *Morpho* butterfly-inspired optical diffraction, diffusion, and bio-chemical sensing†

**DOI:** 10.1039/c8ra04382e

**Published:** 2018-07-30

**Authors:** Rajib Ahmed, Xiaochao Ji, Raghied M. H. Atta, Ahmmed A. Rifat, Haider Butt

**Affiliations:** Nanotechnology Laboratory, School of Engineering, University of Birmingham Birmingham B15 2TT UK a.rajib@osamember.org h.butt@bham.ac.uk; Bio-Acoustic MEMS in Medicine (BAMM) Laboratory, School of Medicine, Stanford University Palo Alto CA 94304 USA; School of Metallurgy and Materials, University of Birmingham Birmingham B15 2TT UK; Electrical Engineering Department, Engineering College, Taibah University Madinah Saudi Arabia; Nonlinear Physics Centre, Research School of Physics and Engineering, The Australian National University Acton ACT-2601 Australia

## Abstract

*Morpho*-butterfly is well-known for the blue colouration in its tiny wing scales and finds applications in colour filters, anti-reflecting coatings and optical devices. Herein, the structural optical properties of the *Morpho peleides*-butterfly wing scales were examined through light reflection, diffraction and optical diffusion. The light diffraction property from wing scales was investigated through experiments and computation modelling. Broadband reflection variation was observed from different parts of the dorsal wings at broadband light illumination due to tiny structural variations, as verified by electronic microscopic images. The periodic nanostructures showed well-defined first-order diffraction through monochromatic (red, green and blue) and broadband light at normal illumination. Polyvinyl alcohol (PVA) embedded with *Morpho peleides*-butterfly wing scales acts as an optical diffuser to produce soft light. Light diffraction and diffusion properties were measured by angle-resolve experiments, followed by computational modelling. The maximum optical diffusion property at ∼185° from the wing scales was observed using broadband light at normal illumination. Finally, *Morpho peleides*-butterfly based submicron nanostructures were utilized to demonstrate bio-inspired chemical sensing.

## Abstract 2

*Morpho* butterfly-inspired structures were used as optical devices (diffraction, diffusion, *etc.*). Their optical performance were modelled and studied, revealing their potential for real-life bio-sensing applications.

## Graphical abstract

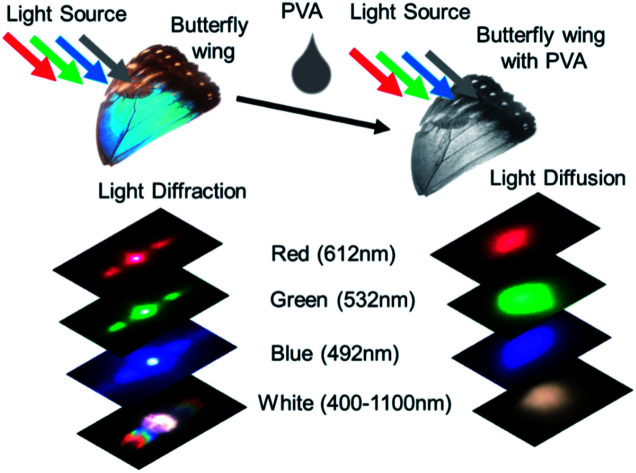

## Introduction

The natural colour of many insects is due to the complex structure of their wings and scales that cause light diffraction, interference, scattering *etc.* at the submicron scale.^1^ Natural species including birds, beetles, flowers, fishes, and seashells show photonic nanostructures that enable bright structural colors.^2^ Moreover, green fluorescent protein (GFP), first isolated from jellyfish, also known as optical highlighters, has found many applications in modern science and medicine.^3^ Natural sub-wavelength optical phenomena based on reflection, diffraction, polarization, and absorption have different applications in displays, bio-chemical sensing, energy harvesting and solar cells.^4–7^ Intricate natural structural colours remain intact and brilliant even after millions of years.^8^ Structural colours are also beneficial for generating bright and stable irradiance over large viewing angles, which contradicts the definition of irradiance definition.^9^ One famous example of structural colour is the blue *Morpho*-butterfly (genus *Morpho* found in Central and South America), whose wing scales produce blue irradiance.^10^ Its structural blue irradiance is due to the alternation between the refractive index (RI) of the air and that of the multi-layered lamina structures or pigments.^11,12^ The layered ridges of chitin that cover its wings are also responsible for the blue irradiance.^13,14^ Highly dense discrete ridges also cause ordered diffraction and additional reflection.^9,15^ Moreover, random offsets among the ridges broaden the sharp reflected/diffracted peaks of multilayer structures, which enable stable colours over larger viewing angles.^16–18^ Strong diffraction and light scattering causes structural colours on the wing scales of the *Morpho* butterfly.^19^ Multiple interferences from the stacked-layer structure are also responsible for strong irradiance.^20–24^ Multi-layered TiO_2_/SiO_2_ was used to mimic the blue irradiance of the *Morpho* butterfly.^25,26^*Morpho* butterfly wing scales were replicated using focused-ion-beam, chemical-vapour-deposition (FIB-CVD), E-beam and laser-interference lithography techniques, which reproduced blue coloration.^27,28^ However, the blue irradiance observed from the chitin/air multilayer structures of blue *Morpho*-butterfly wing scales provide a larger colour gamut and better colour stability compared with fabricated/reproduced structures.^1,29^ Therefore, the bright coloration from blue *Morpho* butterfly wing scales has found advanced applications in displays, self-cleaning surfaces, optical diffusers, ultra-violet sensors, thermal imaging, spectroscopy gas/chemical/vapour sensing, *etc.*^4,5,30–35^

Herein, we report a comprehensive study on the microscopic and optical properties of *Morpho peleides* butterfly wing scales. The optical reflection property of the front and back sides of *Morpho peleides* butterfly wing scales were investigated at blue, brown and black regions. A maximum blue reflection of 3.0% and a minimum black reflection of 0.5% were observed using broadband (white) light at normal illumination. The optical diffraction property was experimentally investigated, followed by computation modelling. First order diffraction was observed with monochromatic, red, green, violet and broadband light illumination at the front and back-side wing scales. At normal illumination, maximum optical diffraction was observed at a wavelength of 635 nm (red light) and the minimum optical diffraction was observed at a wavelength of 450 nm (violet light), which showed similar properties to normal grating structures. *Morpho peleides* butterfly wing scales were also embedded into polyvinyl alcohol (PVA) to act as an optical diffuser, which is able to diffuse light omnidirectionally. In this case, the maximum optical diffusion at about 185° was observed with broadband light illumination. A diffusion angle of approximately 165° was observed with monochromatic light illumination. Moreover, wing scales were embedded into chemical solutions to generate a visual response with refractive index variation. As the refractive index increases, the reflected light shifts from green to blue. Furthermore, the pH and ethanol sensing response was measured to be approximately 5 nm^−1^ and 8.33 nm min^−1^, respectively.

## Results and discussion

### Optical appearances of *Morpho peleides* butterfly wing scales

**Fig. 1:**
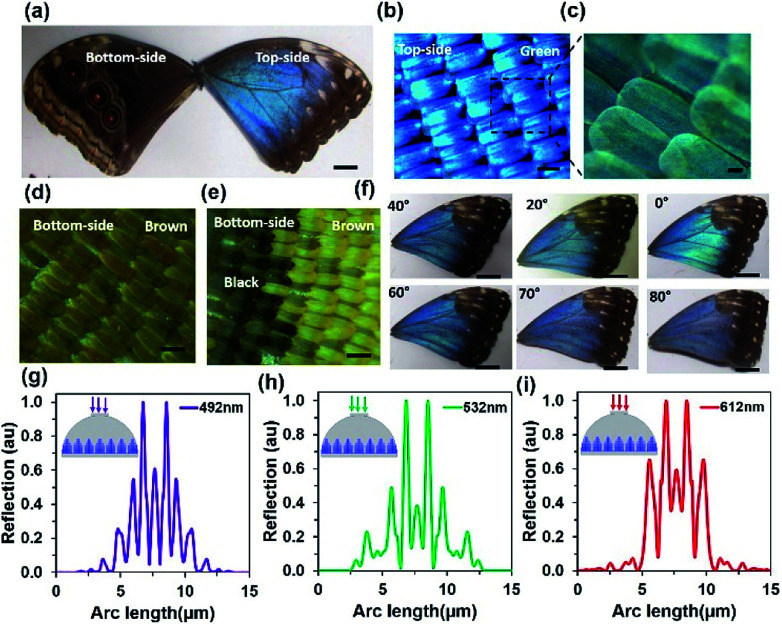
*Morpho peleides*-butterfly-based structural colour. (a) Top-side and bottom-side view of the *Morpho*-butterfly; scale bar = 1.0 cm. (b) Microscopic image of the top-side green structure; scale bar = 200 μm. (c) Magnified version of the top-side green structure. (d and e) Microscopic images of the back-side brown and black-brown structures; scale bars = 200 μm. (f) Structural colours of the *Morpho peleides*-butterfly with tilted surface variation. (g–i) Computation modelling of normalized reflection from the ‘Christmas tree’ structure through monochromatic light illumination (violet, green and red).

The structural colour of *Morpho* butterfly wing scales can be divided into the front-side and the back-side. Dorsal wing scales (the front-side) consist of blue and brown regions, as shown in Fig. 1a (left-side). The central part of the wing scales consist of the green region and is surrounded by the brown region at the edge, as displayed in Fig. 1a (right-side). The ventral wing scales (the back-side) consist of brown and black regions. The edge-side of the wing scales consists of a lighter brown region compared with the central part. However, there are also some black regions (circles) on the centre of the ventral part. Each side consists of brown-ground scales that are uniformly covered. Fig. 1b and c show optical microscopic images of the magnified structure of the front-side blue region. The well-organized green petals of the wing scales in periodic order were observed.

Fig. 1d and e show optical microscopic images of the back-side brown and black-brown regions. The magnified microscopic images of green, brown, and black wing scales are also shown in ESI (Fig. S1†). Randomly positioned black petals of the wing scales were observed and showed lower resolution due to maximum light absorption. Fig. 1f shows the angle dependent colour reflection with a view-angle variation from 0° to 80°. As the view angle increases, the green reflection at 0° changes to a blue reflection at a maximum view-angle of 80°. Therefore, reflected light from the *Morpho peleides*-butterfly wing scales strongly depend on the observer view-angle. Furthermore, the light reflection and diffraction property of the wing scales was computationally modelled, as shown in Fig. 1g–i. The internal ‘Christmas tree’ microscopic structure of the *Morpho* butterfly wing scales has been reported previously.^10,13^ Therefore, light reflection and diffraction from the ‘Christmas tree’ structure were modelled at monochromatic light normal illumination. The reflected light shows multi-order diffraction patterns. As the incident wavelength increases, the diffraction order also increases. Maximum and minimum diffraction orders were observed with violet and red light normal illumination. The diffracted/scattered electric field intensity distribution increased with higher illumination wavelengths (Fig. S2†). Computation modeling of *Morpho* butterfly wing scales has been reported.^36–39^ However, previously reported results were based on broadband light illumination that showed light reflection properties. However, computation modeling of the light diffraction effects from *Morpho peleides* butterfly wing scales for monochromatic light illumination has not been reported. In this study, we modeled monochromatic (612, 532, and 492 nm) diffraction from *Morpho peleides*-butterfly wing scales and the results were comparable to the experimental results. As the monochromatic wavelength increased, the diffracted light scattering angle increased to a larger wavelength.

**Fig. 2:**
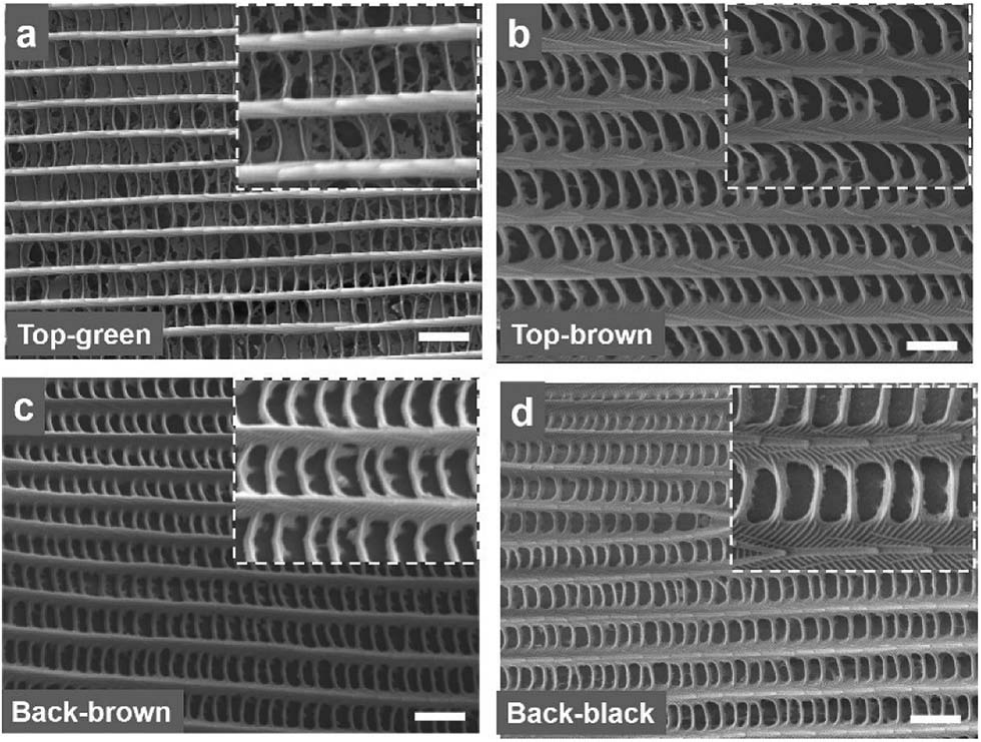
SEM images of the *Morpho peleides*-butterfly wing scales. (a and b) SEM images of the top-side green and brown. Inset images show magnified versions. (c and d) SEM images of the bottom-side brown and black regions. Inset images show magnified versions. Scale bars = 2.0 μm.

Fig. 2 shows SEM images of the distinguished colour regions of the *Morpho* wing scales at the front and back-sides. The microscopic images show wing scale coloration due to the structural variations. The scales found in these regions were compared to front and side-views (Fig. 2a–d and S3†). Dorsal blue wing scales consist of long periodic rigid structures and shorter periodic layers that connect long rigid structures, as shown in Fig. 2a. Each long rigid structure consists of stacked layers (which are better visualized when magnified), as shown in Fig. 2a; the side-view is shown in Fig. S4.† In addition, there are small parallel layers between the stacked layers. The single rigid structure with stack layers is also known as “rigid lamella”. Similar dorsal/ventral wing scales (brown/black) showed rigid stacked layers and a connecting structure between the top and side views, as shown in Fig. 2b–d and S3.† However, the internal structure and wing scales of the blue/brown/black colour of the dorsal/ventral-side are different from the top/side view. Therefore, the coloration of the *Morpho peleides* butterfly wing scales does not depend solely on the pigmentation, but also on the periodic nanostructures.

### Optical reflection on the front-side and back-side of the wing scales

**Fig. 3:**
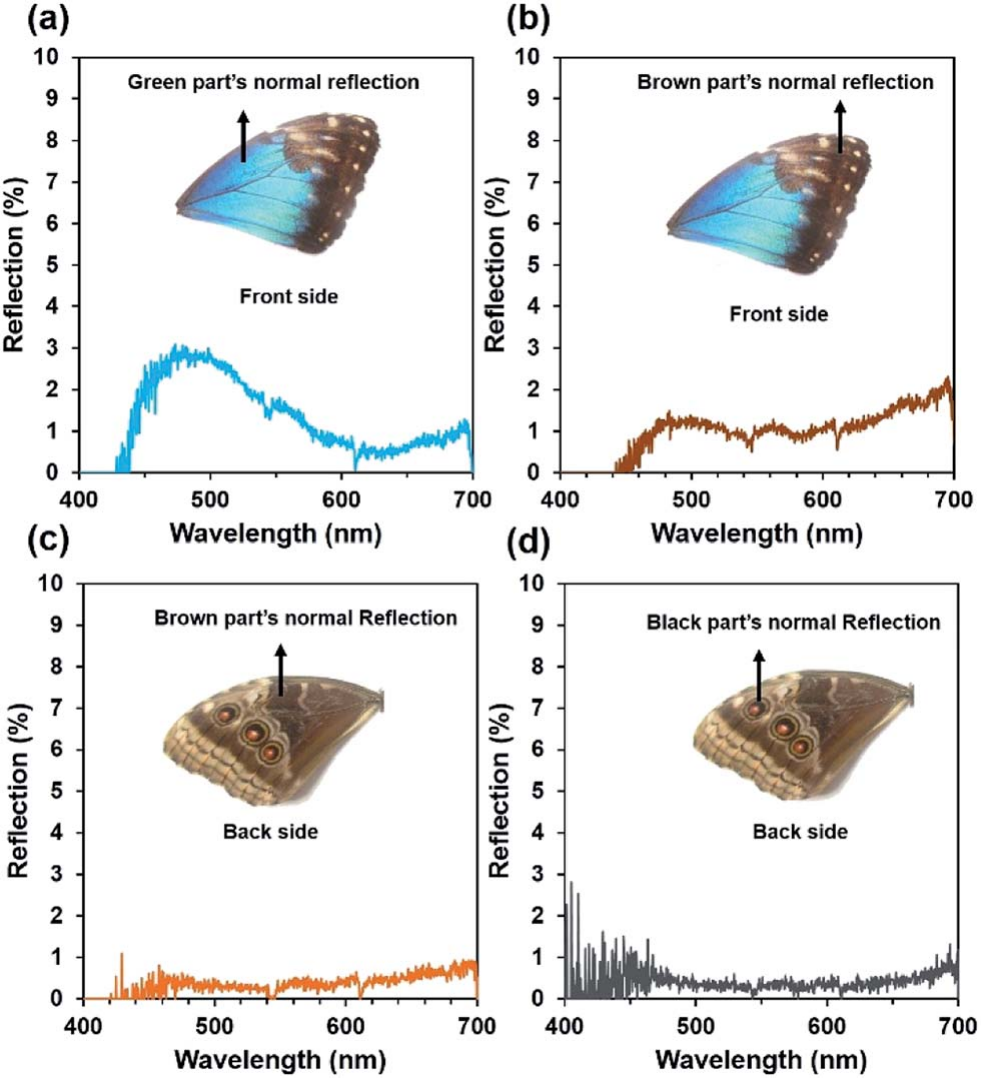
Normal reflection property of *Morpho peleides*-butterfly wing scales. (a and b) Front-side reflection from the green and brown wing scale. (c and d) Back-side reflection from the brown and black wing scale.

Optical reflection property of the blue, brown, and black coloured wing scales was examined on the dorsal/ventral side. The *Morpho peleides*-butterfly wing scales (dorsal/ventral) were illuminated with a broadband light source (450–1100 nm). The reflected light from the wing scales was measured using an Ocean Optics 2000 spectrometer (450–1100 nm, 0.2 nm resolution). The light reflection was measured by an optical microscopic arrangement. For this experiment, light illuminated normal to the wing scales and the reflected light was measured through an objective lens (×20) in reflection mode. Fig. 3a–d show the reflected light intensity from the blue, brown, and black regions on the dorsal/ventral side. Maximum blue reflection was observed in the blue wing scale. The front-side brown wing scales showed higher light reflection compared with that of the back-side brown wing scales. Generally, dorsal wing scales consist of well-ordered petals compared with the ventral wing scales. Therefore, dorsal wing scales show higher reflection than the ventral wing scales. The black region in the back-side shows minimum reflection. This may be due to the different internal multi-grating structure and the randomness of the petal arranged in the black regions that reduces light reflection and increases absorption.

### Optical diffraction based on wing scales

Light diffraction is an optical phenomenon that splits the broadband light waves into rainbow patterns at different directions due to parallel and closely spaced periodic structures of materials. For monochromatic light illumination, periodic grating produces an array of periodic spots, which are perpendicular to the periodic structures. A bright diffraction envelope consists of a high intensity non-diffracted zero-order (*m* = 0) and low intensity higher orders (*m* = ±*n*, *n* = 1, 2, 3…). The optical properties of the diffraction grating have different applications including spectroscopy, wavelength filters, tuneable lasers, displays, holography, and security applications.^40,41^ The optical diffraction property of the *Morpho peleides* butterfly wing scales was observed through light illumination on the dorsal/ventral-side. The wing scales consists of periodic and closely spaced periodic cuticle ridge structures. The ridge consists of periodic lamella thin films.

**Fig. 4:**
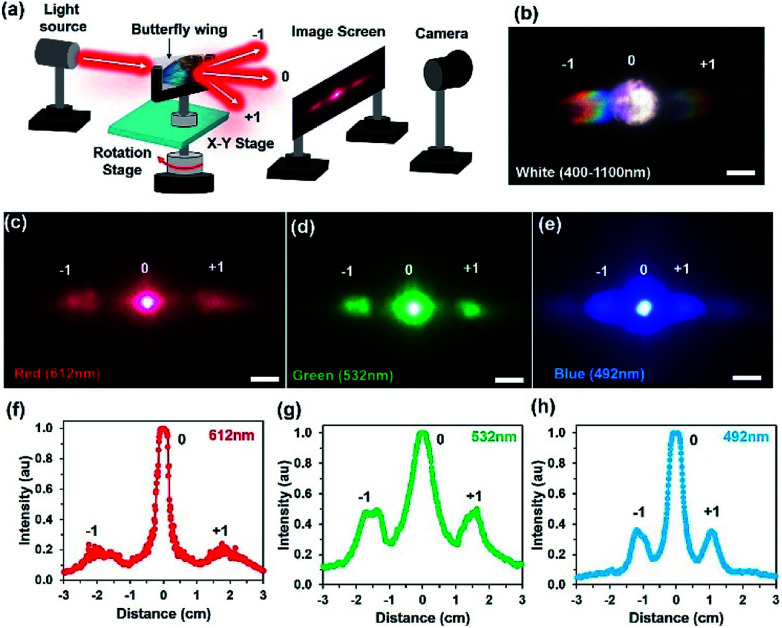
Optical diffraction property of *Morpho peleides*-butterfly wing scales. (a) Conceptual block diagram of far-field diffraction with the image-screen setup. (b–e) Far-field diffraction through monochromatic and broadband light at normal illumination. Scale bars = 1.5 cm. (f–h) FFT simulation of the far-field diffracted intensity through monochromatic light illumination.

Light diffraction occurs due to the periodic structural variations of the ‘ridge-lamella’ inside the *Morpho* butterfly wing scales. Fig. 4a shows the image-screen experiment set-up to observe the optical diffraction property of the blue *Morpho peleides*-butterfly wing scales. Light illuminated at the butterfly-wing scales and far-field diffracted light were observed on an image-screen. A digital camera was used to capture the diffraction patterns at the low intense mode. For broadband light illumination, the wing scales showed rainbow patterns (Fig. 4b). Moreover, first-order diffraction patterns were also observed through monochromatic light illumination (Fig. 4c–e). The broadband (white) and monochromatic (red, green, and violet) light were illuminated normal to the ventral-side of the wing scales. However, similar optical diffraction properties were also observed from illuminated light at the dorsal-side.

Fast Fourier Transform (FFT) simulation of the far-field diffraction patterns was performed for the monochromatic light illumination. The first-order diffraction distance of the far-field patterns was calculated for red, green, and violet light illumination to be 3.8, 3.12 and 2.3 cm, respectively (Fig. 4f–h). The diffraction distance increases as the wavelength increases, following Bragg's laws:1
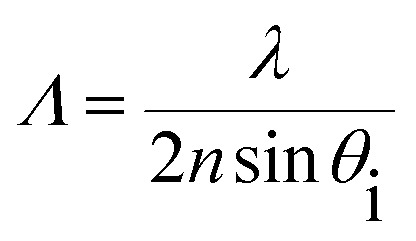
where *Λ* is the grating spacing, *λ* is the incident wavelength, *n* (= 1,2,3…) is an integer, and *θ*_i_ is the incidence angle.^42^ The green light shows maximum diffraction intensity with normalized light illumination.

**Fig. 5:**
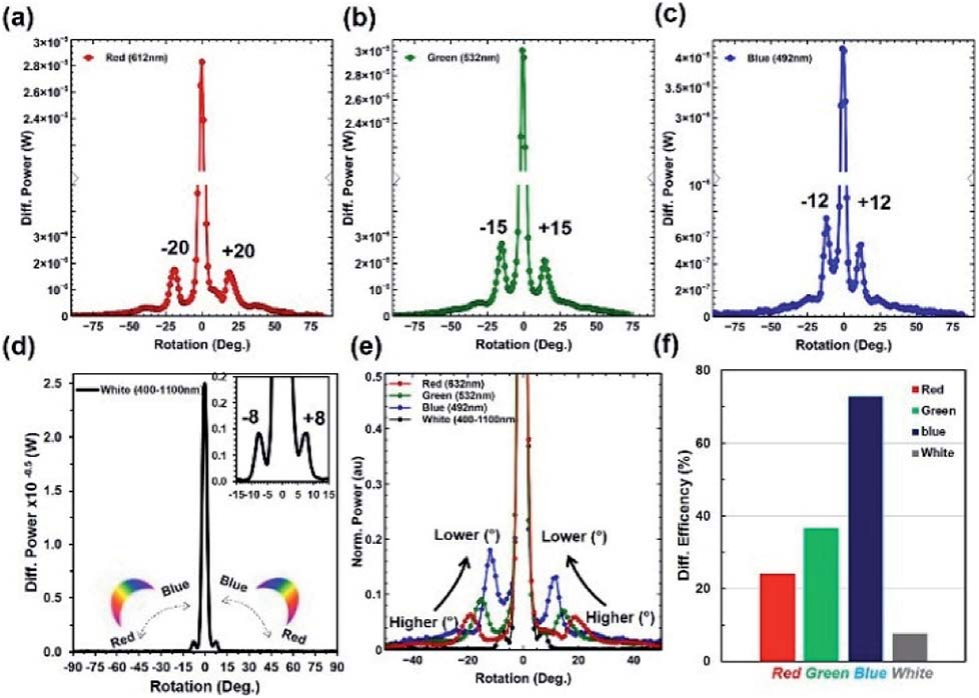
Diffraction grating property of *Morpho peleides*-butterfly wing scales. (a–e) Diffraction power intensity as a function of rotational angle (°) through light illumination. (f) Diffraction efficiency with monochromatic (red, green and blue) and broadband light normal light illumination.

Furthermore, the light diffraction property was measured through an angle-resolve setup.^43^ The *Morpho peleides* butterfly wing sample was attached to a glass slide and fixed through a sample holder. The sample was positioned through a 2D *x*–*y* stage and rotated 360° (step size = 1°) through a microcontroller operated motorized stage. The incident light was normally illuminated and light diffraction was measured through an optical power meter. The step size of the rotational stage was controlled and power meter readings were stored and plotted in real time through microcomputer-based customized software. Fig. 5a–e show the measured diffraction power as a function of rotation angle through monochromatic and broadband light illumination. Well-defined first-order diffraction patterns match the simulated far-field projection results. The first-order diffraction of red, green, violet, and white light were observed at rotational positions of 20, 15, 12, and 8°. As the wavelength decreases, the first diffraction peak shifts from higher to lower values (Fig. 5f). Maximum and minimum diffraction peak distances with respect to non-diffracted zero-order were observed through the red and white light illumination. The diffraction efficiency (DE) is the ratio between the diffracted and incident light power and expressed as a percentage. Maximum and minimum diffraction efficiencies were observed through blue and white light normal illumination (Fig. 5f). Therefore, *Morpho peleides*-butterfly wing scale-based nanostructures can be used as wavelength selective diffractive elements.

### Optical diffusion based on wing scales

**Fig. 6:**
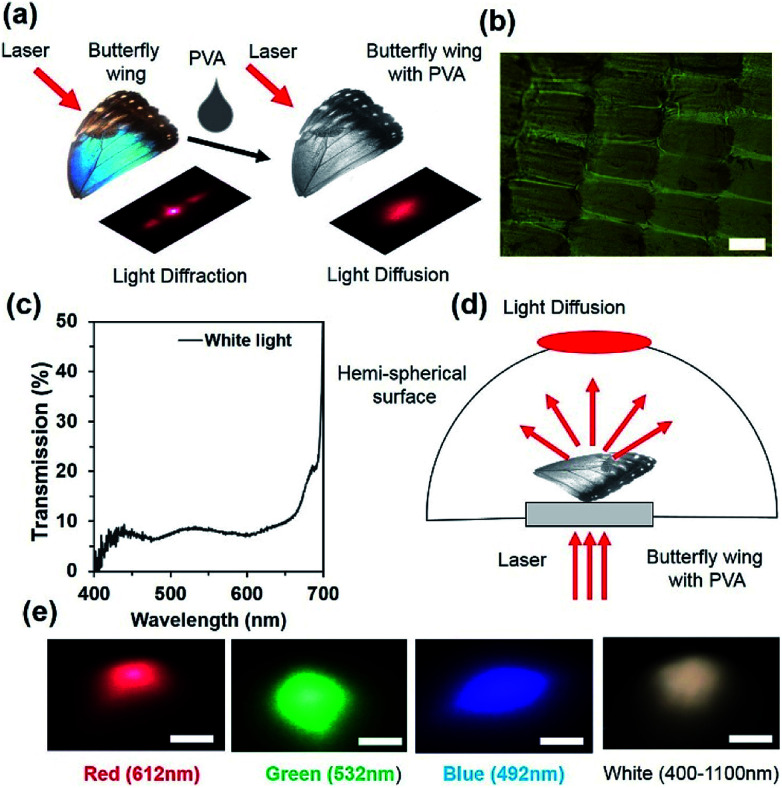
*Morpho peleides*-butterfly wing scale-based optical diffuser. (a) Conceptual diagram of optical diffuser formation through PVA embedded with the *Morpho* wing scale. (b) Optical microscopic image of the PVA embedded with *Morpho* wing scales. Scale bar = 100 μm. (c) Optical transmission property of the PVA-*Morpho* wing scales diffuser. (d) Conceptual diagram of far-field optical diffuser captured through a hemispherical setup. (e) Far-field diffraction measurement through monochromatic and broadband light illumination. Scale bars = 4 cm.

An optical diffuser is an optical device that produces soft or scattered light from a highly intense focused light. The light diffusion property is due to the surface-relief microstructures or surface roughness that redistributes light waves. Optical diffusers have applications in car flash/head lights, lamps, light emitting diodes (LEDs) and displays. Optical diffusers have been produced through complex lithography and e-beam techniques.^41^ However, direct laser written optical glass diffusers were also fabricated through a femtosecond and nanosecond laser.^41,44^ Ink-based optical diffusers were also reported recently using a single-pulsed nanosecond laser and reflection holography.^41^ However, they have been limited with smaller diffusion angle. *Morpho didius* butterfly-inspired cholesteric liquid crystals were used to demonstrate circular polarized light diffusion and the diffusion angle was limited between 80 and 100°.^2^ However, in this study, we have demonstrated that *Morpho peleides* butterfly wing scales embedded in PVA were used to modify the photonic bandgap and enable unpolarized light diffusion over a broad range (diffusion angle ∼185°). A few drops of liquid PVA was poured in *Morpho* butterfly wing scales. Light diffraction property of the wing scales was converted into light diffusion property when the *Morpho peleides* butterfly wing was covered by a thin layer of PVA, as shown in Fig. 6a. Light diffusion occurs as the gaps between the periodic and closely spaced internal ridge-lamella structures of the wing scales are filled, as shown in Fig. 6b. The transmission property of the PVA-*Morpho* butterfly wing scales was measured through an optical microscopic arrangement. Broadband light transmission was observed over the visible range (Fig. 6c).

The light diffusion property of the PVA *Morpho*-wing scales was also examined through a semi-transparent hemispherical surface (Fig. 6d). When monochromatic (red, green and violet) and broadband (white) light were illuminated normal to the PVA-*Morpho* butterfly wing scales through a glass plate, a diffused light at the hemispherical surface was captured through a digital camera at low intensity mode. Fig. 6e shows the captured far-field diffused patterns with monochromatic and broadband light illumination. The far-field patterns showed a well-distributed/spread light over larger hemispherical areas, which proves that the PVA-*Morpho* butterfly wing can work as a good diffuser surface.

**Fig. 7:**
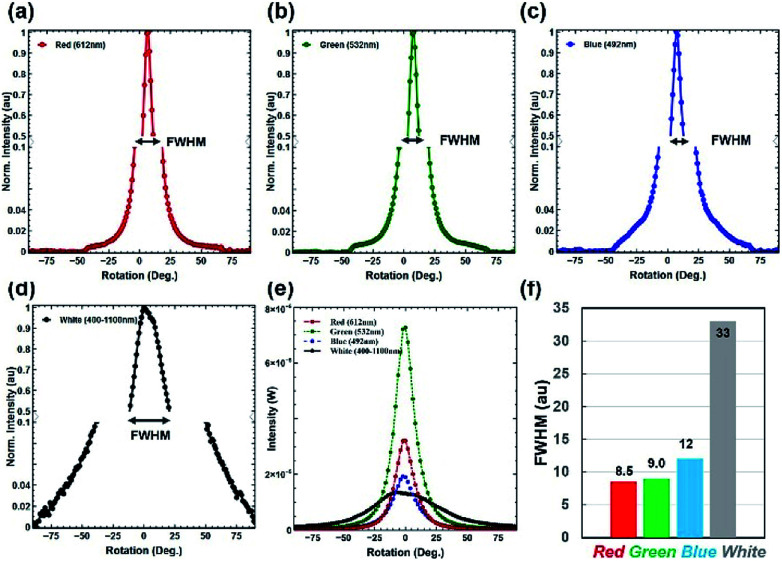
*Morpho peleides* butterfly-based optical diffuser spectrum characterization. (a–e) Diffused light intensity as a function of rotation angle (°). (f) FWHM of the diffused red, green, blue and white light source.

Furthermore, optical diffusion property measurements were conducted using the angle-resolved motorized rotational stage, as previously explained. Monochromatic (red, green and violet) and broadband (white) light were used to illuminate the PVA-*Morpho* butterfly wing scale sample and the diffused light was measured through a power meter (Fig. 7a–e). Tilted illumination was varied from −90° to +90° with a 1° rotational step size. A wide diffusion angle of about 125° (−50° to +75°) was observed with monochromatic light illumination. Moreover, an extreme diffusion angle of 180° was also observed with broadband light illumination. This is the highest diffusion angle observed compared to previously reported optical diffusers.^41,44^ Therefore, maximum FWHM was observed with broadband light illumination (Fig. 7f).

### *Morpho peleides*-butterfly-based bio-chemical sensors

**Fig. 8:**
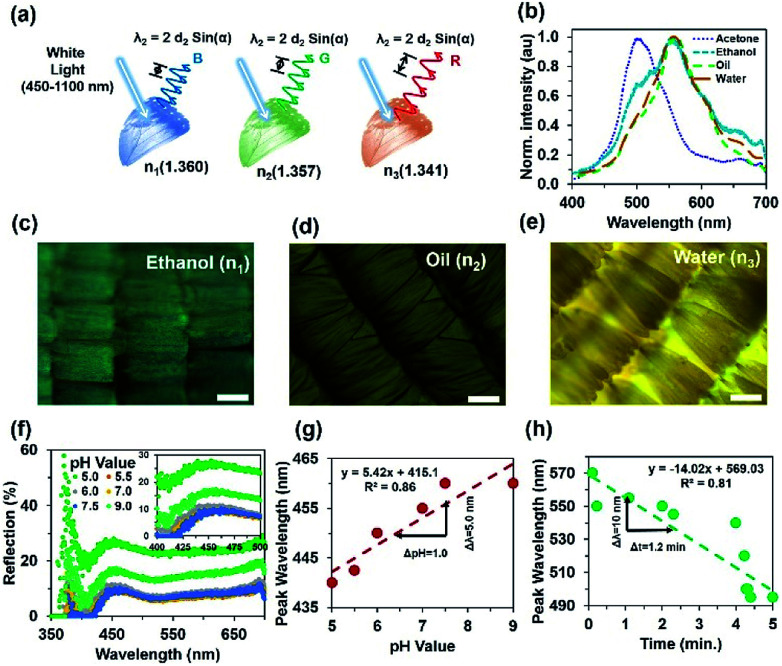
*Morpho peleides*-butterfly-based bio-chemical sensing. (a) Conceptual sensing principle based on RI variation. (b) Reflected peak wavelength shifting due to RI variation (acetone, ethanol, oil, water) through broadband light illumination. (c–e) Microscopic image of PVA-based IPN through ethanol, oil and water immobilization. Scale bars = 100 μm. (f) Light reflection through pH (5.0–9.0) variation. (g) Reflected peak-wavelength as a function of pH variation. (h) Reflected peak wavelength as a function of time due to an ethanol immobilized wing sample.

*Morpho peleides* butterfly wing scales were individually embedded into PVA as an interpenetrating polymer network (IPN) to generate a visual response on refractive index variation. The peak-wavelength of the reflected light from the immobilized wing scales depends on the internal grating structure variations due to RI variation of the swelling/de-swelling of IPN. Fig. 8a shows the conceptual diagram for the *Morpho peleides* butterfly wing scale-based sensing principle. The reflected/diffracted light is shifted to the left/right with swelling/shrinking of the IPN network. Fig. 8b shows the reflected light intensity with acetone (*n* = 1.3600), ethanol (*n* = 1.3573), oil (*n* = 1.3410) and water (*n* = 1.330) immobilization. The refractive indices of the solutions were measured through an analog refractometer (KERN, ORA 80 BB).

The reflection experiment with normal broadband light illumination (90° incident and reflection angle) was performed using a spectrophotometer and optical microscope setup. White light from the microscope's external source was used to illuminate the surface of the immobilized wing scales (through the ×20 objectives) and reflected back to the spectrophotometer (top of the microscope). Microscope objectives were used to collimate the light beam, which was focused onto the sample and measured with a spectrophotometer. Optical sensing performance also depends on the size of the beam. Higher resolution objectives (×50, ×100) provide smaller spot sizes and reduce the reflected light intensity. Therefore, the shift in peak wavelength due to analyte variations was difficult to observe. For the ×20 objective, the light focusing area increased by several scales and an average sensing response was observed over a large area.

Fig. 8c–e show optical microscopy images of the *Morpho peleides* butterfly wing scale-embedded IPN. The swelled IPN wing scales showed bight green as well as dark and light brown colours with ethanol, oil and water immobilization. Uniformity may vary with samples positions and reduce the sensing performance. Therefore, the best wing-scales with good uniformity were identified by microscopic imaging before sensing. The reflected light intensity with change in pH (5.0–9.0) of the immobilized *Morpho peleides* butterfly wing scales showed slight shifts in peak wavelength (Fig. 8f and g). As pH increased, the reflected peak value shifted towards higher wavelength. Fig. 8g shows the linear curve for the shift in peak wavelength as a function of pH. Shifts in wavelength occur both in acidic (pH < 7) and alkaline (pH > 7) solutions. The maximum peak wavelength at 440 nm shifted to 442.5, 450, 455, and 460 nm as pH changed from 5.0 to 9.0. Furthermore, the reflection property of the acetone immobilized *Morpho peleides* butterfly wing scales showed a maximum blue reflection at a peak wavelength of 502 nm (Fig. 8h).

As the refractive index value decreases, the peak wavelength shifts to the right. The maximum blue peak at 502 nm shifts to 556, 558 and 560 nm as broadband light reflects from ethanol, oil and water immobilized wing scales, respectively. Furthermore, a sensing experiment was performed with ethanol-immobilized *Morpho* butterfly wing scales with different time duration. With passage of time, the colour of the immobilized wing scales will change. Therefore, the reflected colour will change according to the wing's colour. The reflected colour shifts to the left (smaller wavelength) due to shrinking of IPN with longer observation time. At longer time periods, most of the ethanol solution evaporates from the *Morpho peleides* butterfly wing scales surface due to its volatile nature; therefore, reflected/diffracted light shows smaller peak wavelengths.

Fig. 8h shows the reflected peak wavelength as a function of observation time. The peak wavelength shifts linearly towards shorter/longer wavelength with increased/decreased time or with shrinking/swelling of IPN. Therefore, immobilized *Morpho peleides* butterfly-based ethanol sensing can be measured from the linear tangent of the response curve. The peak wavelength of the normal illuminated reflected light depends on pH, observation time, and environmental conditions as follows:2
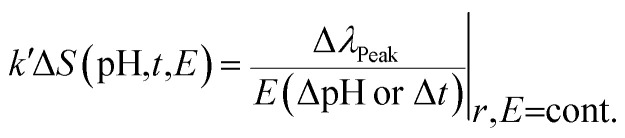
where *k*′ is the proportionality constant, which is related to the environmental conditions (temperature, relative humidity, *etc.*), Δ*S*(pH,*t*,*E*) is the change in sensitivity as a function of pH variation, observation duration (*t*) and environment conditions (*E*). Therefore, the sensitivity, *S*, is the ratio between the peak wavelength shift, Δ*λ*_Peak_, and pH variation, ΔpH, or observation duration, Δ*t*. At fixed sample position, *r*, and environment condition, *E* (23 °C, 34% RH), the pH and ethanol sensitivity, *S*, were found to be approximately 5.0 nm^−1^ and 8.33 nm min^−1^, respectively. The sensing property based on the swelling and shrinking condition of the hydrogel is due to different pH or RI variation.^45^ The volume of the hydrogel IPN and variation in RI can influence the wing scale sub-micron structures and in turn, change the light reflection property and shift the wavelength. Therefore, left/right shifting of the reflected peak wavelength (blue/red) depends on swelling/shrinking of the hydrogel and RI variation. The main component of the butterfly wings is chitin and the embedded butterfly wing-scale with PVA is soft and fragile. Therefore, they are not suitable for pH sensing in a severe environment.

## Conclusions

In summary, we demonstrate that different colour regions of *Morpho peleides* butterfly wing scales depend not only on the pigmentation, but also on the periodic nanostructures inside the wing scales. The structural colour of the wing scales is strongly dependent on the observer's viewing angle. As the viewing angle increased from 0 to 80°, the reflected colour shifted from blue to violet. Optical diffraction was observed with monochromatic/broadband light normal illumination on the dorsal/ventral-side of the wing scales. Well-defined first order diffraction patterns were observed at far-field due to periodic lamella/ridge structures. *Morpho peleides* butterfly wing scales embedded into PVA and acted as an optical diffuser that diffused light omnidirectionally. The maximum diffusion angle (∼185°) was observed with broadband light illumination. Finally, wing scales embedded with chemical solutions acted as a refractive index sensor that produced a visual response. Therefore, *Morpho peleides* butterfly wing scales have potential applications as diffractive/deflective nanophotonics and bio-chemical sensing devices.

## Materials and methods

### Sample preparation, microscopic imaging and reflection measurement

PVA, acetone and ethanol were purchased from Sigma-Aldrich, USA. PVA samples were prepared by dissolving 20 g of PVA in 100 g of distilled water by stirring for 2 hours at 60 °C using a magnetic stirrer integrated with an electric heater (StableTemp hot plates, purchases from Cole-parmer, UK). Air bubbles were then removed from the PVA solution using an ultrasonic bath (GT Sonic) and a vacuum chamber. To prepare different buffer solutions with pH values ranging from 5.0–9.0, HCl and NaOH were diluted with distilled water and the pH value was measured with a pH meter (PHS-3C, Shanghai Precision and Scientific Instrument).

Naturally dead *Morpho peleides* butterflies were collected from the local market and dried samples under glass plates were prepared. Scales were removed from the wings, and their microscopic images were taken using an optical microscope (AXIO Scope. A1, Carl Zeiss Microscopy GmbH, Germany).

*Morpho peleides* butterfly wings were first cleaned with acetone then dried with air. The prepared PVA solution was poured into the butterfly wing to fill the gaps between the nanostructures. The sample was tilted to make a thin layer and dried with hot air before light illumination. To avoid the charging effect of the non-conductive butterfly wings, the SEM wing scale samples were coated with a thin platinum layer for 30 s each using a sputter coater (Quorum Technologies Ltd), and then characterized by a JEOL 7000 scanning electron microscope (SEM) with a working distance of 10 mm and an accelerating voltage of 5 kV, as shown in Fig. 2a–d. Normal reflection from different parts of the wing scales were measured using an optical microscope (integrated with a CCD camera at the top) in reflection mode. A spectrophotometer with a resolution of 0.1 nm and a 450–1100 nm broadband light source (purchased from Ocean Optics) were used for optical measurements. FFT simulations and data processing were performed with MATLAB (MathWorks, v8.1).

### Computation modeling

To understand the light diffraction property from the ‘Christmas tree’ structure, computational modelling was performed with the finite element methods (FEMs) using a commercial COMSOL multi-physics simulation tool.^42^ Scattering and periodic boundary conditions were considered during the 2D simulation. Triangular mesh elements were considered during FEM computation. The computed mesh consists of 1183 boundary elements, 126 884 degrees of freedom, and 17 963 domain elements. Total computation time was about 20 seconds. A convergence test was conducted to access the accuracy of the results.

### Optical characterization

Optical diffraction and diffusion properties of butterfly wing scales were characterized using a spectrophotometer with broadband and monochromatic light sources. Angle-resolved measurements were performed using a customized setup. Broadband and monochromatic light was normally illuminated onto the sample, which was kept fixed in a sample holder. The sample was rotated from 0 to 180° through microcontroller-based rotational stages. The diffracted light was measured through a power meter (PM100D) and sensor (S120C), which were purchased from Thorlabs Elliptec GmbH (Dortmund, Germany) and the data values were stored and plotted auto-metrically through a microcomputer. Far-field diffraction experiments were performed using a hemispherical surface with *Ø* = 30 cm and an image screen setup using white A4 paper. The monochromatic light sources, namely, red (612 nm, 4.5 mW, *Ø*11 mm), green (532 nm, 4.5 mW, *Ø*11 mm), and blue (492 nm, 2.6 mW, *Ø*11 mm), were purchased from Thorlabs Elliptec GmbH (Dortmund, Germany).

## Conflicts of interest

There are no conflicts to declare.

## Supplementary Material

RA-008-C8RA04382E-s001
