# Supplementary material for: Morpho butterfly-inspired optical diffraction, diffusion, and bio-chemical sensing
Source: RSC Adv. 2018 Jul 30;8(48):27111–8. doi: 10.1039/c8ra04382e (PMC9083500; doi:10.1039/c8ra04382e)
Supplement: RA-008-C8RA04382E-s001 [file RA-008-C8RA04382E-s001.pdf]

Electronic Supplementary Material (ESI) for RSC Advances.
This journal is © The Royal Society of Chemistry 2018

Supplementary Information

Morpho-Butterfly Inspired Optical Diffraction, Diffusion, and Bio-Chemicals Sensing

Rajib Ahmed,[1,2] Xiaochao Ji,[3] Raghied M. H. Atta,[4] Ahmmed A. Rifat,[5] and Haider Butt [1,*]*

[1] Nanotechnology Laboratory, School of Engineering, University of Birmingham, Birmingham B15 2TT, United Kingdom

[2] Bio-Acoustic MEMS in Medicine (BAMM) Laboratory, School of Medicine, Stanford University, Palo Alto, CA 94304, USA

[3] School of Metallurgy and Materials, University of Birmingham, Birmingham B15 2TT, United Kingdom

[4] Electrical Engineering Department, Engineering College, Taibah University, Madinah, Saudi Arabia

[5] Nonlinear Physics Centre, Research School of Physics and Engineering, The Australian National University, Acton, ACT-2601, Australia

*Correspondence: a.rajib@osamember.org , h.butt@bham.ac.uk

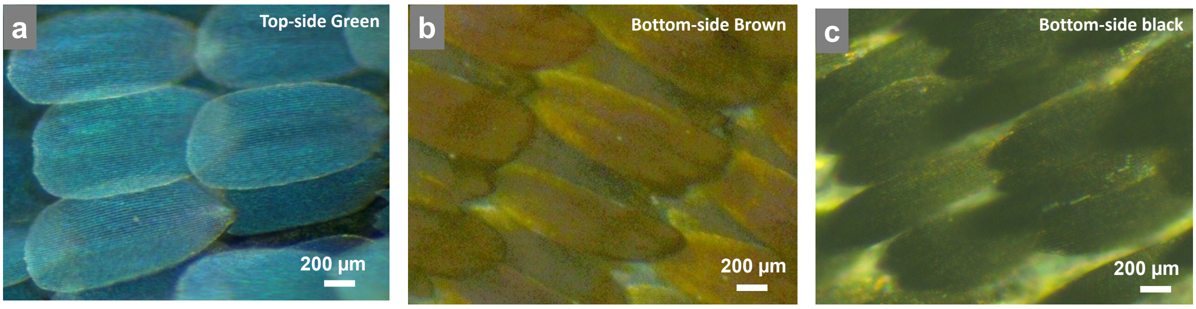

Figure S1: Magnified microscopic image of *Morpho*-butterfly wing scale. (a) Top-side green, (b,c) Bottom-side brown and black regions.

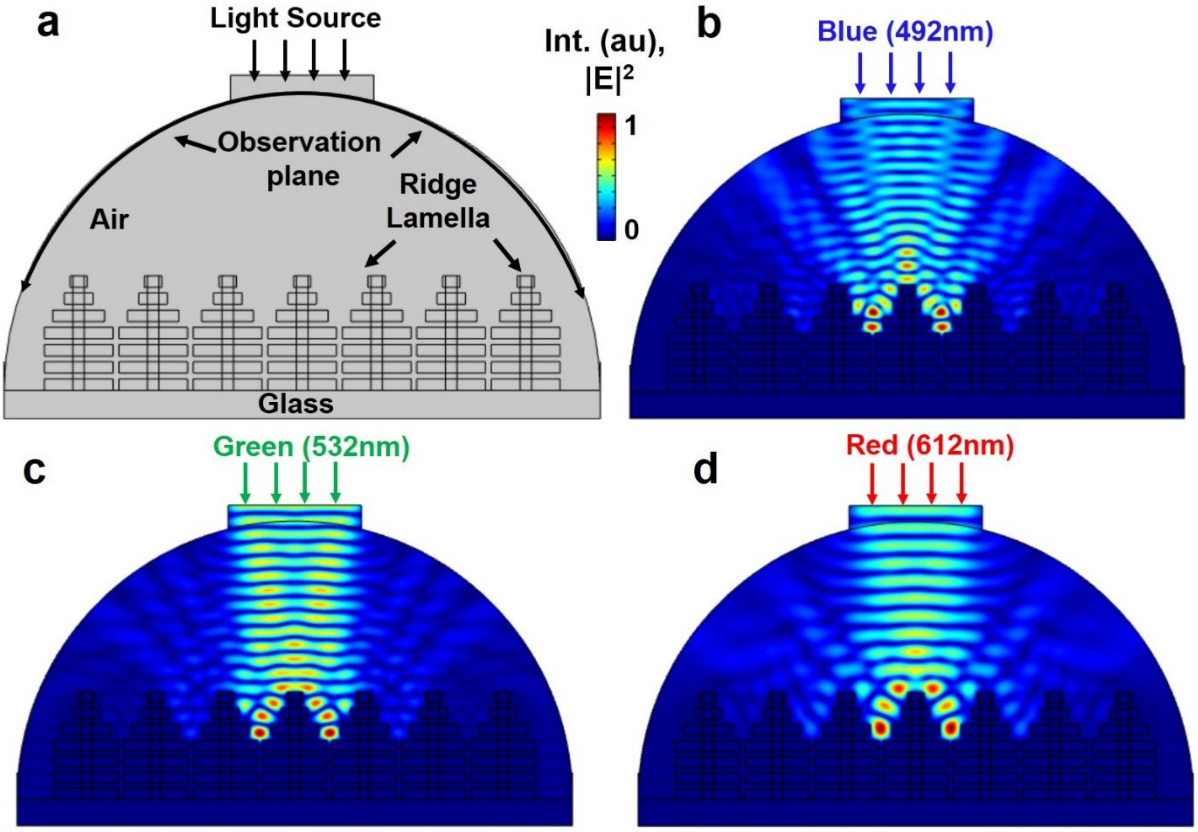

Figure S2: (a) Computational block diagram of normal reflection from ridge lamella structure. (b-d) Electric field's intensity distribution with violet, green, and red light normal illumination.

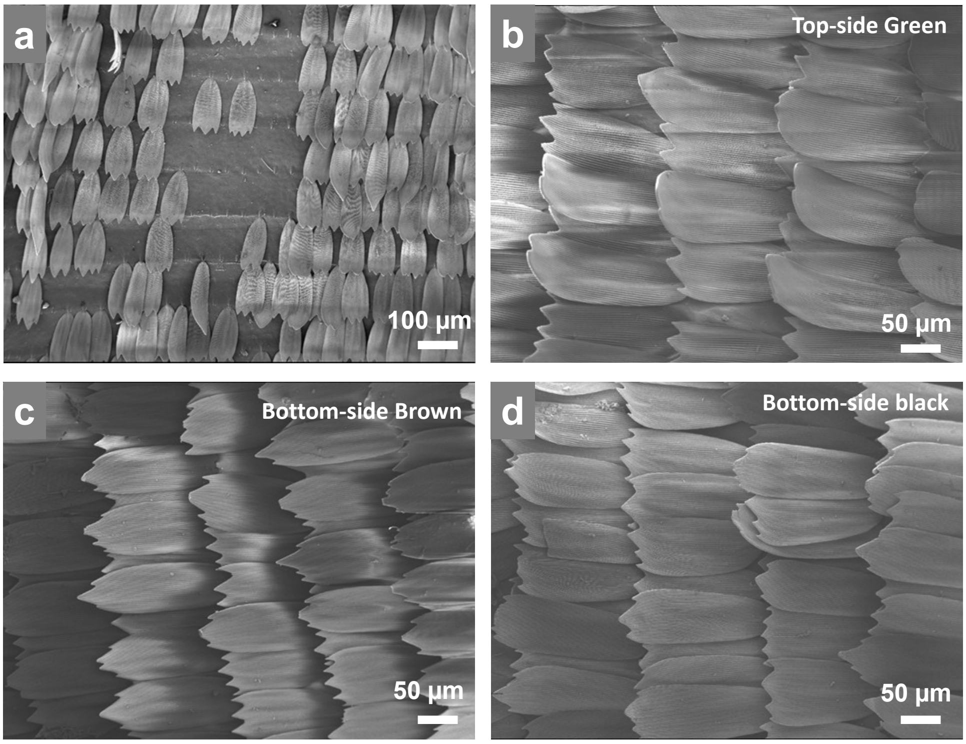

Figure S3: SEM image of *Morpho*-butterfly wing scale. (a) Layered structure, (b-d) Top-side green, (b,c) bottom-side brown and black regions.

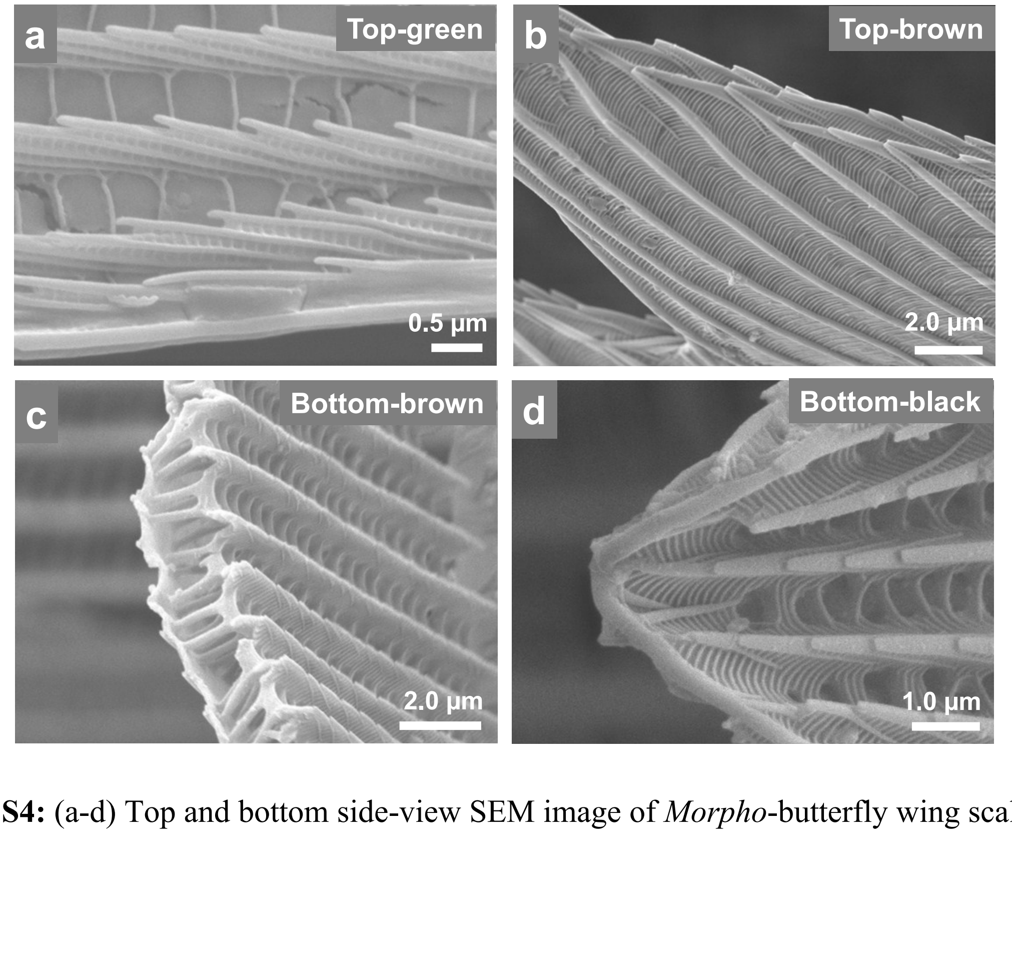

Figure S4: (a-d) Top and bottom side-view SEM image of *Morpho*-butterfly wing scale.
